# Supplementary material for: Correlates of SARS-CoV-2 Breakthrough Infections in Kidney Transplant Recipients Following a Third SARS-CoV-2 mRNA Vaccine Dose
Source: Vaccines (Basel). 2025 Jul 22;13(8):777. doi: 10.3390/vaccines13080777 (PMC12389881; doi:10.3390/vaccines13080777)
Supplement: Supplementary file 1 [file vaccines-13-00777-s001.zip › vaccines-3715715-supplementary.pdf]

Supplementary materials

Figure S1. Total numbers of SARS-CoV-2 infections in the general population in Denmark over time. The bar chart is combined with our study timeline illustrating vaccine dose administrations and data sampling of our study cohort.

Figure S2. Proportion of SARS-CoV-2 variants of concern (VOC) dominating in the general population in Denmark over time. The graph is combined with our study timeline illustrating vaccine dose administrations and data sampling of our study cohort.

Figure S3. Antibody-mediated neutralization (%) against selected variants of SARS-CoV-2, stratified by immunosuppressive regimen. CNI, Calcineurin inhibitor. Antimetabolites MMF, mycophenolate mofetil, and MPA, mycophenolic acid. (mean; SEM).

Figure S4. Venn diagram showing combinations of immunosuppressive regimen.
